# Landscape of Health-Related Quality of Life in Patients With Early-Stage Pancreatic Cancer Receiving Adjuvant or Neoadjuvant Chemotherapy: A Systematic Literature Review

**DOI:** 10.1097/MPA.0000000000001507

**Published:** 2019-03-10

**Authors:** Teresa Macarulla, Andrew E. Hendifar, Chung-Pin Li, Michele Reni, Hanno Riess, Margaret A. Tempero, Amylou C. Dueck, Marc F. Botteman, Chinmay G. Deshpande, Eleanor J. Lucas, Do-Youn Oh

**Affiliations:** From the ∗ Gastrointestinal and Endocrine Tumor Unit, Vall d'Hebron University Hospital and Vall d'Hebron Institute of Oncology, Barcelona, Spain; † Division of Hematology and Oncology, Cedars-Sinai Medical Center, Los Angeles, CA; ‡ Division of Gastroenterology and Hepatology, Department of Medicine, Taipei Veterans General Hospital; § Department of Medicine, National Yang-Ming University School of Medicine, Taipei, Taiwan; ∥ Department of Medical Oncology, IRCCS Ospedale San Raffaele, Milan, Italy; ¶ Department of Medicine, Charité-Universitätsmedizin Berlin, Freie Universität Berlin, Humboldt-Universität zu Berlin, Berlin Institute of Health, Berlin, Germany; # Division of Hematology and Oncology, University of California, San Francisco, CA; ∗∗ Helen Diller Comprehensive Cancer Center, San Francisco, CA; †† Alliance Statistics and Data Center, Mayo Clinic, Scottsdale, AZ; ‡‡ Real-World Evidence and Data Analytics, Pharmerit International, Bethesda, MD; §§ Department of Internal Medicine, Seoul National University Hospital, Cancer Research Institute, Seoul National University College of Medicine, Seoul, South Korea.

**Keywords:** health-related quality of life, minimally important difference, pancreatic cancer, pancreatic resection, patient-reported outcome measures

## Abstract

**Objectives:**

Pancreatic resection is associated with postoperative morbidity and reduced quality of life (QoL). A systematic literature review was conducted to understand the patient-reported outcome measure (PROM) landscape in early-stage pancreatic cancer (PC).

**Methods:**

Databases/registries (through January 24, 2019) and conference abstracts (2014–2017) were searched. Study quality was assessed using the Newcastle-Ottawa Scale/Cochrane risk-of-bias tool. Searches were for general (resectable PC, adjuvant/neoadjuvant, QoL) and supplemental studies (resectable PC, European Organisation for Research and Treatment of Cancer QoL Questionnaire [QLQ] – Pancreatic Cancer [PAN26]).

**Results:**

Of 750 studies identified, 39 (general, 22; supplemental, 17) were eligible: 32 used QLQ Core 30 (C30) and/or QLQ-PAN26, and 15 used other PROMs. Baseline QLQ-C30 global health status/QoL scores in early-stage PC were similar to all-stage PC reference values but lower than all-stage–all-cancer values. The QoL declined after surgery, recovered to baseline in 3 to 6 months, and then generally stabilized. A minimally important difference (MID) of 10 was commonly used for QLQ-C30 but was not established for QLQ-PAN26.

**Conclusions:**

In early-stage PC, QLQ-C30 and QLQ-PAN26 are the most commonly used PROMs. Baseline QLQ-C30 global health status/QoL scores suggested a high humanistic burden. Immediately after surgery, QoL declined but seemed stable over the longer term. The QLQ-C30 MID may elucidate the clinical impact of treatment on QoL; MID for QLQ-PAN26 needs to be established.

## Abstract 2

Supplemental digital content is available in the text.

Surgical resection is the only potentially curative option for pancreatic cancer (PC).^1,2^ However, pancreatic resection is associated with significant postoperative morbidity and reduced quality of life (QoL).^3–5^ In early-stage PC, adjuvant chemotherapy may significantly improve survival outcomes compared with surgery alone,^6,7^ and recent trials of combination chemotherapy are changing the treatment landscape.^8–10^ Current guidelines of the National Comprehensive Cancer Network, American Society of Clinical Oncology (ASCO), and European Society for Medical Oncology (ESMO) recommend adjuvant chemotherapy as a standard of care for patients with resected PC.^1,2,11^ In addition, some studies have documented conversion of locally advanced, unresectable PC to resectable status with neoadjuvant chemotherapy, and the postsurgical survival rates in these patients were comparable to those observed in patients with resectable disease.^12–14^ It is not completely clear how recent advances have influenced QoL in patients with early-stage PC who have undergone surgery and received chemotherapy. Therefore, it is important to understand the landscape of patient-reported outcome measures (PROMs) that can quantify and track QoL in these patients.

In advanced PC, tools such as the European Organisation for Research and Treatment of Cancer (EORTC) Quality of Life Questionnaire Core 30 (QLQ-C30), the EORTC Quality of Life Questionnaire Pancreatic Cancer Module (QLQ-PAN26), and the EuroQoL 5-Dimension Questionnaire (EQ-5D) have been used to assess the effect of therapy on QoL.^15–17^ However, in early-stage PC, it is unclear from the few available studies of QoL which PROMs are most commonly used and appropriate. A more thorough understanding of QoL in early-stage PC, including how it changes over time after surgery as well as the PROMs most commonly used to measure QoL, may help identify specific areas that have not been fully examined and help clinicians use appropriate symptom management and adjuvant strategies in this patient population.

To address this need, we conducted a systematic literature review (according to Preferred Reporting Items for Systematic Reviews and Meta-Analyses [PRISMA] guidelines) of studies evaluating QoL in patients with early-stage PC who underwent resection to (1) assess the landscape of the QoL PROMs used, (2) understand how QoL changes over time after surgical resection, and (3) assess which specific thresholds have been used to define a minimally important difference (MID) in QoL.

## MATERIALS AND METHODS

### Search Strategy and Study Selection

A team composed of medical oncologists, health economics and outcomes research scientists, and a statistician formed a panel to develop the search, selection, and review strategies. Databases (Medline, Embase [via ProQuest], Cochrane Database of Systematic Reviews, and Cochrane Central Register of Controlled Trials) and registries (ClinicalTrials.gov and International Clinical Trials Registry Platform) were searched through January 24, 2019. In addition, congress abstracts and presentations (ASCO, ASCO Gastrointestinal Cancers Symposium, and ESMO World Congress of Gastrointestinal Cancer) from 2014 to 2017 were searched. Studies were included in accordance with the PRISMA statement.^18^ Search terms were designed to include specific populations (resectable or borderline-resectable PC), interventions (adjuvant or neoadjuvant chemotherapy), and outcomes (QoL as assessed by PROMs; Supplemental Table 1 http://links.lww.com/MPA/A776). Any of the following study designs were permitted: randomized, controlled trials (phase 2, 3, or 4); single-arm trials; observational studies; prospective studies; and protocols (for the MID and PROM identification objectives). Only studies published in English were considered. The original search did not identify any study reporting MID results for the EORTC QLQ-PAN26 PROM; therefore, a supplemental search with expanded search terms (resectable or borderline-resectable PC; EORTC QLQ-PAN26) was conducted to identify studies that assessed MID for EORTC QLQ-PAN26 (Table, Supplemental Digital Content 1 http://links.lww.com/MPA/A776). Studies of advanced PC, case series, case reports, nonsystematic literature reviews, nonhuman studies, and studies with no abstract were excluded. Duplicates were removed, and only the most up-to-date reports of research were included (eg, congress presentations were removed if a peer-viewed article was identified).

### Data Extraction

Two reviewers independently screened all titles and abstracts to develop a list of reports for full-text review. Any discrepancies were adjudicated by a third reviewer. Reports selected for full-text review were screened by 1 reviewer for data extraction and qualitative synthesis. Data on population characteristics (eg, location, time frame, sample size, and demographic characteristics), interventions (eg, adjuvant or neoadjuvant chemotherapy), and outcomes (eg, PROMs used, survival data, QoL [including longitudinal data, when available], and MID) were extracted from the included studies into a database.

### Assessment of Study Quality

The study quality was assessed independently by 2 reviewers, and a third reviewer resolved any disagreements. Nonrandomized observational studies were assessed using the Newcastle-Ottawa Scale (NOS),^19^ and randomized controlled trials were assessed using the Cochrane risk-of-bias tool.^20^ The NOS assesses study quality in 3 domains—selection, comparability, and outcome—and assigns scores of ≤4, 2, and 3 points, respectively, yielding a total maximum score of 9. A study was considered to be of high quality if the total NOS score was ≥7.^21,22^ The Cochrane risk-of-bias tool assesses study quality in 6 bias domains: selection (sequence generation and allocation concealment), performance (blinding of participants and personnel), detection (blinding of outcome assessments), attrition (incomplete outcome data assessment), reporting (selective reporting), and other (any important concern not covered in the other domains). The Cochrane tool assigns a risk of bias—low, high, or unclear—for each category.

### Result Synthesis

In a narrative synthesis, the EORTC QLQ-C30 global health status (GHS)/QoL scale scores were compared with EORTC QLQ-C30 reference norms^23^ and assessed longitudinally when possible. The MID estimates for the most frequently used PROMs were assessed.

## RESULTS

### Study Selection and Data Extraction

**FIGURE 1:**
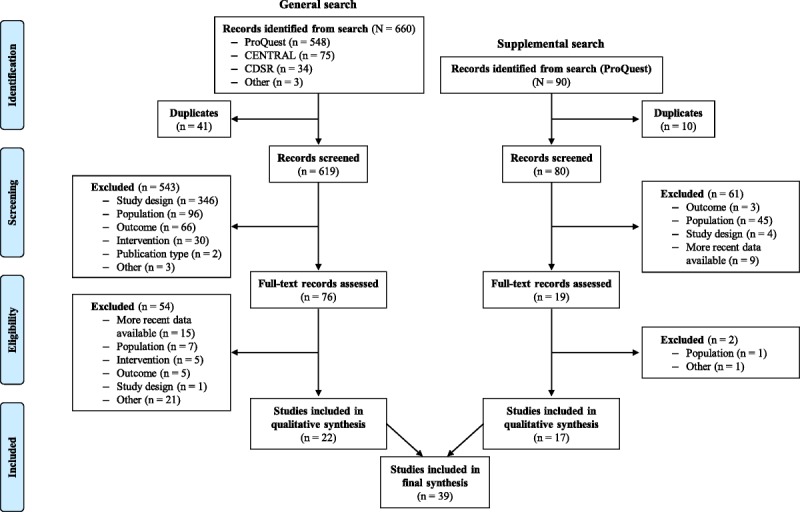
Flow diagram based on PRISMA. CDSR, Cochrane Database of Systematic Reviews; CENTRAL, Cochrane Central Register of Controlled Trials.

Of the 750 records identified initially, 660 were captured from the general search; the supplemental search produced 90 additional records (Fig. 1). After removing duplicates and excluding records during initial screening, 95 studies were assessed in full; of these, 56 studies did not meet the eligibility criteria and were excluded. Overall, 39 studies (22 captured from the general search and 17 from the supplemental search) were included in the final qualitative synthesis (Fig. 1); of these, 28 were observational studies and 11 were randomized, controlled trials (Table 1).

**TABLE 1:**
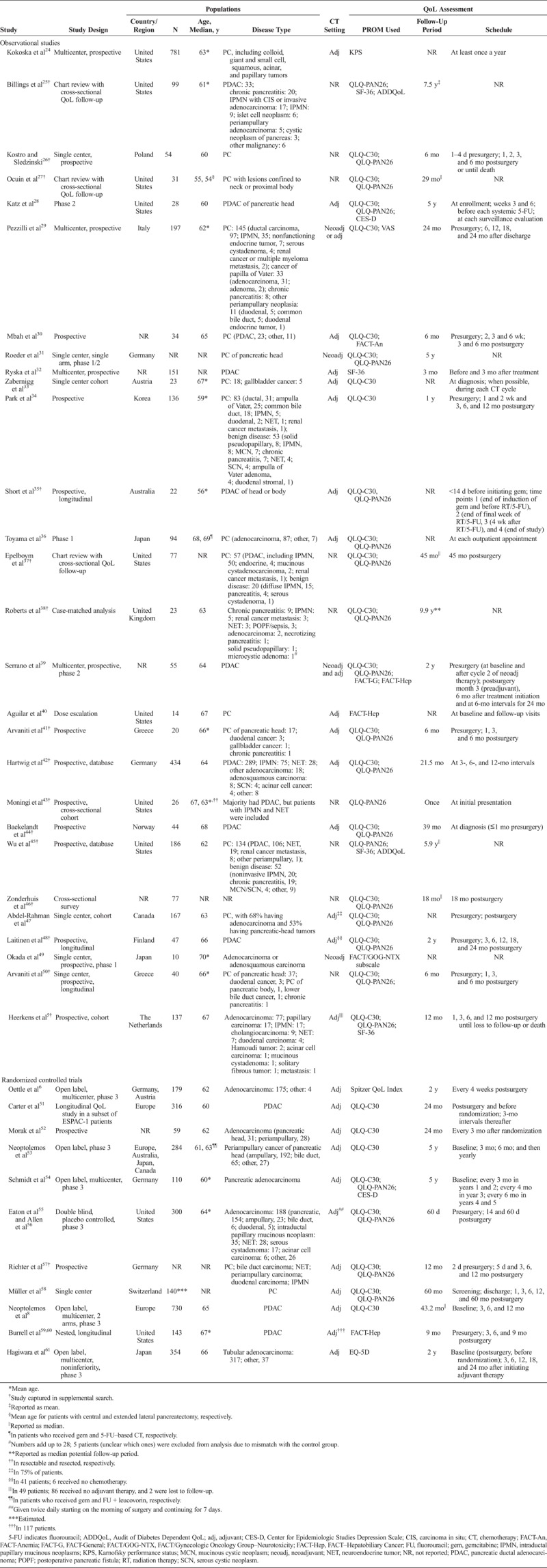
Study Population Characteristics

### Study and Population Characteristics

The key characteristics of the included studies and their respective populations are shown in Table 1. The sources for 3 studies were conference abstracts,^32,46,47^ and the remaining 36 were full journal articles, including 3 study protocols.^31,57,58^ The studied populations included patients in North America, Europe, and Asia who typically received neoadjuvant and/or adjuvant chemotherapy and were assessed for QoL for a variable period (a few months to several years).

**FIGURE 2:**
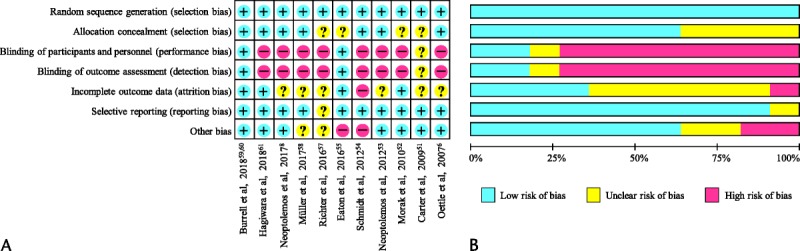
Quality of randomized, controlled trials as assessed by the Cochrane risk-of-bias tool. A, Assessments by study and category. B, Proportion of studies with low, unclear, and high risk of bias in each category.

Using NOS, 11 of the 28 observational studies were assigned a score of ≥7 (high quality), 13 received a score of 5 or 6 (moderate quality; primarily due to comparability and outcome biases), and 4 received a score of 3 or 4 (low quality due to biases in all 3 domains; Table 2). The risks of bias in the 11 randomized, controlled trials as assessed by the Cochrane tool are shown in Figures 2A and B.

**TABLE 2:**
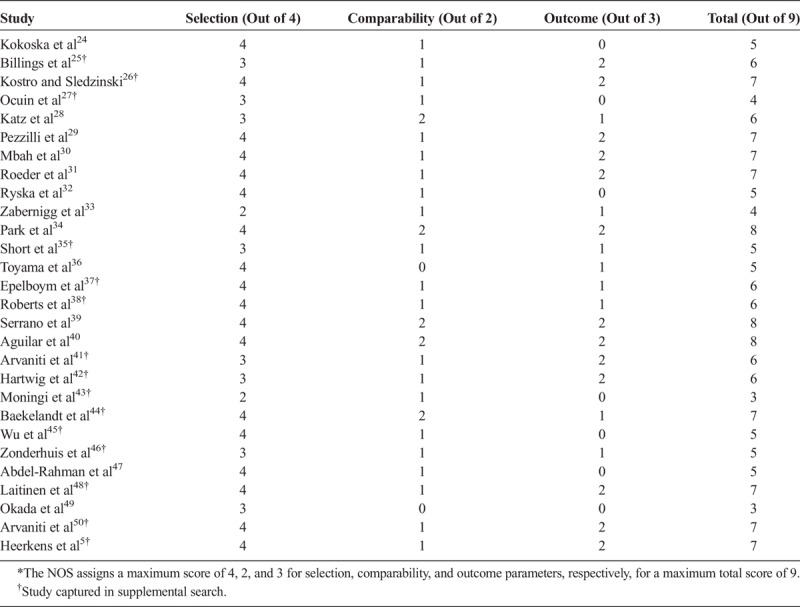
Quality of Nonrandomized Observational Studies as Assessed by Scores on the NOS*

### QoL PROMs Landscape in Early-Stage PC

Among the 22 studies included from the original general search, EORTC QLQ-C30 and QLQ-PAN26 were the most commonly used PROMs (15 [68%] studies); among all 39 studies included from the original and supplemental searches, 32 (82%) used EORTC QLQ-C30 and/or QLQ-PAN26 (Table 1). Overall, 15 studies (nonexclusive) used other PROMs: Functional Assessment of Cancer Therapy (FACT; n = 5), 36-item Short Form Survey (SF-36; n = 4), Center for Epidemiologic Studies Depression Scale (n = 2), Audit of Diabetes Dependent QoL (n = 2), EQ-5D (n = 1), Karnofsky performance status (n = 1), visual analog scale (VAS) for pain (n = 1), and Spitzer QoL Index (n = 1; Table 1). The study that used Karnofsky performance status to assess QoL collected self-reported assessments via mail-in or telephone interview.^24^

### QoL Outcomes

The EORTC QLQ-C30 GHS/QoL scores at baseline were compared with previously reported data for PC or all cancers. The baseline QoL was defined using presurgical data (n = 6) or the first postsurgical data (n = 5). The baseline EORTC QLQ-C30 GHS/QoL scores in early-stage PC (median [interquartile range], 61 [59–64]) seemed similar to reference norms reported for PC (all stages and including liver and biliary cancers; 58 [42–75]; n = 750) but lower than those reported for all cancers (all stages; 67 [50–83]; n = 23,553).^23^

Overall, 13 studies reported longitudinal QoL data; of these, 11 studies reported longitudinal EORTC QLQ-C30 GHS/QoL data (Table 3). Most of these studies used chemotherapy (with or without radiation) in the adjuvant setting (Table 3). Chemotherapy included gemcitabine, FOLFIRINOX (leucovorin, fluorouracil, irinotecan, and oxaliplatin), mitoxantrone, fluorouracil, cisplatin, carboplatin, oxaliplatin, capecitabine, paclitaxel, and pasireotide. In addition to the EORTC QLQ-C30, 6 studies used EORTC QLQ-PAN26 and 5 studies used other PROMs (SF-36, EQ-5D, Pain VAS, and FACT-General/FACT-Hepatobiliary Cancer Subscale) to report longitudinal QoL data. As expected, the number of patients who completed the QoL questionnaires decreased over time in most studies (Table 3).

**TABLE 3:**
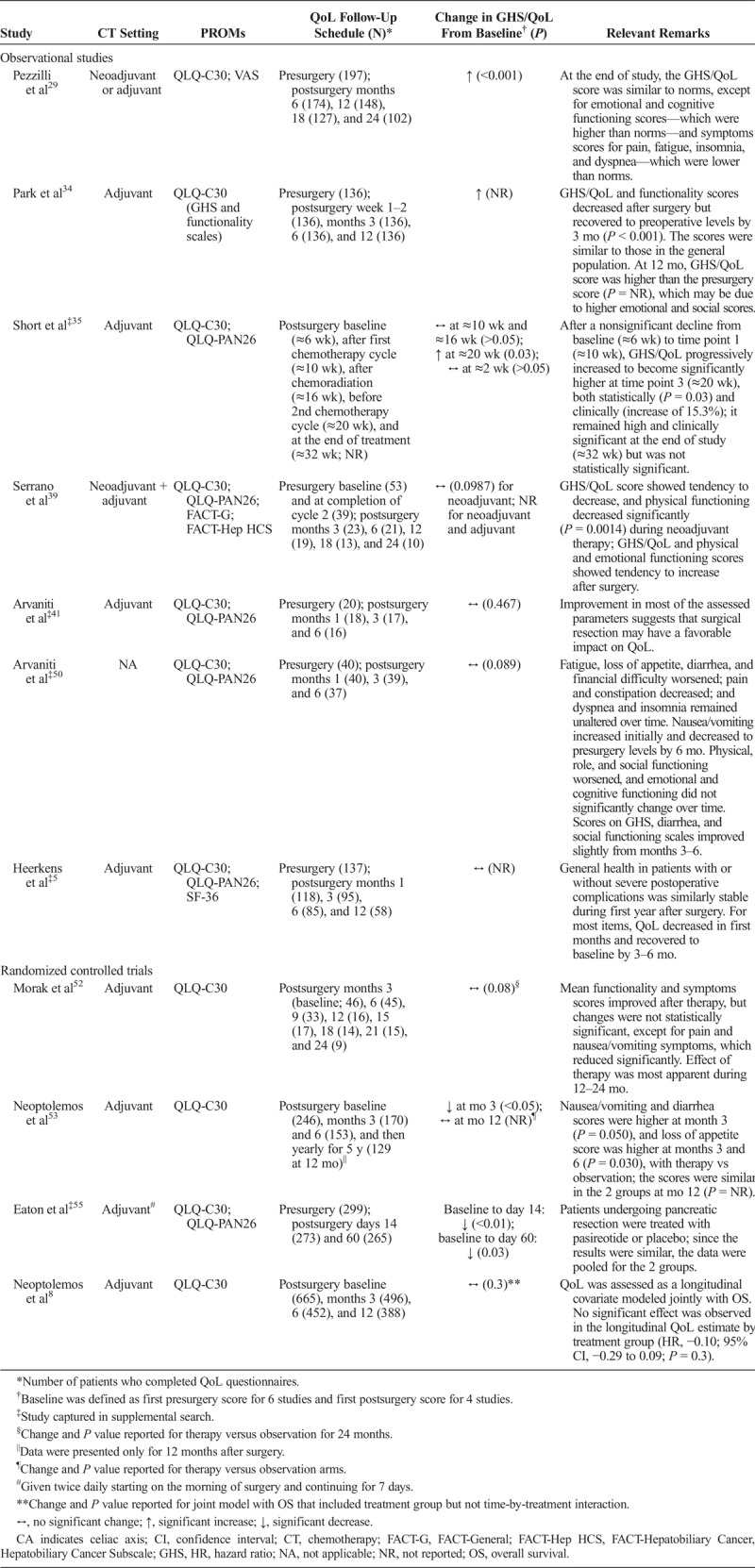
QoL Outcomes Over Time

**FIGURE 3:**
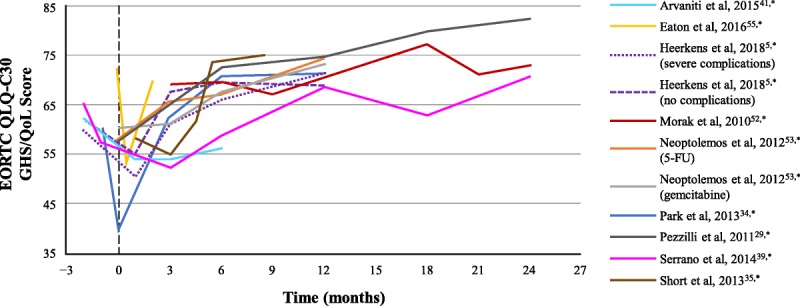
Longitudinal GHS/QoL scores before and after surgery. Dashed vertical line represents the time of surgery. *Study captured in supplemental search. 5-FU, 5-fluorouracil.

Two of the 11 studies that reported longitudinal EORTC QLQ-C30 GHS/QoL data reported change over time but did not report absolute scores; the remaining 9 studies reported GHS/QoL scores in 11 settings at baseline and at multiple postsurgical time points (Fig. 3). Some of these studies reported a transient decline immediately after surgery in GHS/QoL scores, which recovered to baseline levels in 3 to 6 months (Fig. 3, Table 3). In one study, a significant decrease was reported in GHS/QoL scores at 2 weeks (*P* < 0.01) and 2 months (*P* = 0.03) in postsurgical versus presurgical scores.^55^ In another study, the overall QoL scores were significantly lower in patients receiving chemotherapy (5-fluorouracil plus leucovorin [*P* = 0.03] or gemcitabine [*P* = 0.001]) versus observation at 3 months after surgery, but the scores were similar at 12 months after surgery (Table 3).^53^ Collectively, these studies demonstrated a trend of decline in GHS/QoL scores during the first few months after surgery with recovery of GHS/QoL scores over time. Consistent with this observation, Park et al^34^ reported numerically higher GHS/QoL scores at 12 months after surgery versus before surgery, and Pezzilli et al^29^ reported a significant increase in QoL for 24 months after surgery (*P* < 0.001). Most studies (7/10) reported no statistically significant change in GHS/QoL scores over the follow-up period (Table 3). Changes in the EORTC QLQ-C30 functionality and symptoms scales were generally similar to those in the GHS/QoL scale, and no specific patterns were observed in individual scales across studies (data not shown). In the 6 studies that used EORTC QLQ-PAN26, changes in specific subscales varied but were generally consistent with those in QLQ-C30 scales (data not shown).

### MID Outcomes

Six studies used specific thresholds to define clinically important differences in EORTC QLQ-C30 GHS/QoL scores within or between groups (Table 4). Four of these studies used a cutoff of 10 points in QoL scores to define an MID, including 1 study that additionally defined smaller (5–10 points) and larger (>20 points) cutoffs. Two studies used 0.5 × baseline standard deviation (SD) as the cutoff.

**TABLE 4:**
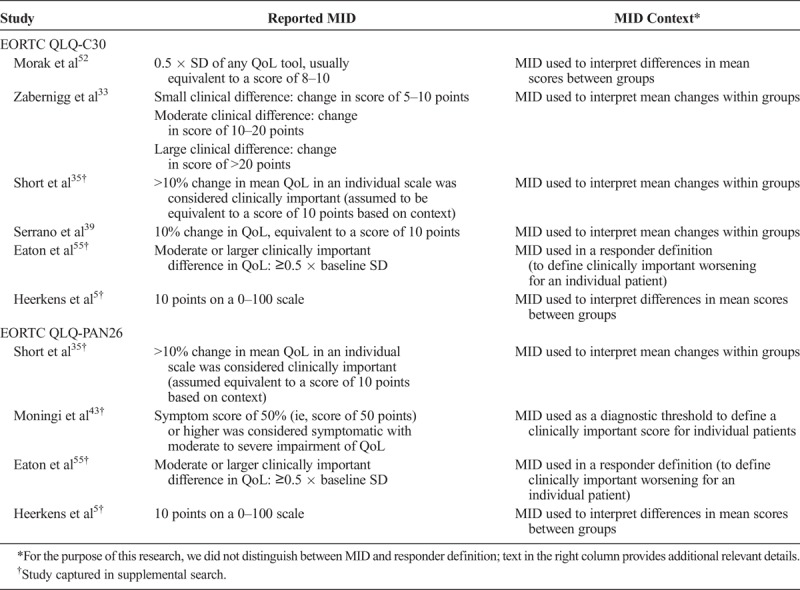
Reported MID Outcomes for EORTC QLQ-C30 and QLQ-PAN26 PROMs

Four studies used specific thresholds to define clinically important differences in EORTC QLQ-PAN26 scores within or between groups (Table 4); of these, 2 studies used 10 points, 1 used 0.5 × baseline SD, and 1 used a specific absolute score to define a clinically important change in QoL. The reported MIDs for EORTC QLQ-C30 and QLQ-PAN26 were used in various ways, including to interpret differences in mean scores between groups, mean changes over time within groups, individual patient scores at a single time point, and individual patient changes over time (ie, to define which patients had clinically important changes; Table 4).

## DISCUSSION

In this systematic literature review assessing QoL PROMs in early-stage PC, EORTC QLQ-C30 and QLQ-PAN26 were identified as the most commonly used QoL PROMs. The EORTC QLQ-C30 GHS/QoL scores at baseline were consistent with reference norms for PC but lower than those for all cancers collectively, supporting the high humanistic burden (ie, challenges faced by patients and their families/caregivers) and unmet need for these patients. The present systematic literature review is one of the few that comprehensively assessed longitudinal QoL data before and after pancreatic resection. The change in QoL over time after surgery varied across the 11 studies that reported these data, but the overarching observation was that QoL initially declined after surgery as observed previously,^53,55^ recovered in approximately 3 to 6 months after surgery, and remained generally stable for the rest of the follow-up period. The QoL dynamics reported here are generally consistent with those from a recent systematic literature review that assessed the effect of pancreatoduodenectomy on QoL (17 studies published up to June 2016; 1240 patients), which showed no change in global health and overall QoL during 12 postoperative months in 6 of the 12 studies; the remaining 6 studies reported a postoperative decline in QoL that recovered after 3 to 6 months.^62^ Similar trends were seen in physical and social functioning domains and in pain, fatigue, and diarrhea symptoms scales.^62^ The initial decline in QoL is consistent with a delayed initiation of adjuvant chemotherapy in a considerable proportion of patients with resected PC,^63,64^ and the recovery and stability suggest that QoL may not be negatively affected by chemotherapy, at least in the longer term. Additional research is needed to understand the effect on QoL of other important factors, such as use of neoadjuvant chemotherapy and intensity of adjuvant chemotherapy.

The MID for EORTC QLQ-C30 and QLQ-PAN26 may be useful in understanding the clinically relevant impact on QoL of treating early-stage PC. An MID of 10% (equivalent to a score of 10 points) change in mean QLQ-C30 scores was considered to be clinically important in most studies. This was generally consistent with the previously reported mean differences between groups (range on different subscales, 4–11) or those over time (improvement, 4–8 points; deterioration, 5–13 points).^65,66^ Two studies also used the same threshold for EORTC QLQ-PAN26 scales, but overall, the MID data for this PROM were limited. Two studies for EORTC QLQ-C30 and 1 for EORTC QLQ-PAN26 used 0.5 × baseline SD as a threshold for identifying clinically important change in QoL. For EORTC QLQ-C30, this threshold seems equivalent to a change of approximately 8 to 10 points in mean scores^52,55^; for EORTC QLQ-PAN26, this threshold may be slightly higher (approximately 10–15 points).^55^ A recent study underscored the unavailability of reference values for EORTC QLQ-PAN26 in the United States,^45^ further suggesting that MID has not been assessed comprehensively and that there is a need to fully establish the reference values for this PROM. The systematic literature review by van Dijk et al^62^ did not assess MID values.

Both EORTC QLQ-C30 and QLQ-PAN26 seem to be useful PROMs in assessing QoL in early-stage PC. However, because EORTC QLQ-PAN26 is specifically designed to assess QoL in patients with PC, it may provide more relevant data to help physicians effectively manage symptoms and make treatment decisions in this patient population. Additional research is needed to further validate the EORTC QLQ-PAN26 PROM and establish the MID for adjuvant and neoadjuvant chemotherapy in early-stage PC.

Understanding QoL in patients with early-stage PC may help improve management strategies after pancreatic resection. Adjuvant chemotherapy improves survival outcomes and is recommended by the National Comprehensive Cancer Network, ASCO, and ESMO guidelines as a standard of care^1,2,6–11^; however, only approximately 50% of all patients undergoing pancreatic resection receive adjuvant therapy.^63,64^ Pancreatic resection is associated with significant postsurgical morbidity and impaired QoL,^3–5^ and postsurgical complications are associated with significantly lower rates and delayed administration of adjuvant therapy.^63,64^ Hence, there may be reluctance among physicians and patients toward adjuvant chemotherapy, which could prompt considerations of neoadjuvant or perioperative treatment. A recent study showed that patients with PC who were undergoing resection experienced high levels of depression before surgery through 6 months after surgery; the study suggested that managing physical symptoms and providing psychological support before surgery may improve QoL outcomes in these patients.^67^ Results from this systematic literature review may guide more efficient management of patients with early-stage PC who are receiving adjuvant chemotherapy and thus improve the overall outcomes in these patients.

### Study Limitations

The studies included in this analysis were heterogeneous in terms of study design (populations and interventions [including the type of chemotherapy]) and QoL assessments (frequency, follow-up duration, and schedule). The reference norms with which the early-stage PC QoL outcomes from this study were compared are approximately a decade old and include all stages of cancer, but these types of data are generally limited in availability, and the norms used here are, to our knowledge, the only such currently available. As a result of disease recurrence, treatment withdrawal, or death, longitudinal QoL assessments do not include the entire initial patient population; therefore, improvement in QoL observed in some studies may reflect survivor selection bias. The changes in QoL over time are presented here in terms of statistical significance, which may not always align with changes of clinical significance. Some did not assess QoL before surgery, which makes it difficult to assess the extent of QoL recovery to the presurgical levels. To partially address this limitation, the graphs across studies were normalized to the time of surgery, which helped to standardize and clarify the trajectory of scores over time. The quality of included studies, as assessed by the NOS and Cochrane risk-of-bias tools, was generally low; however, their collective use in this analysis allowed for a comprehensive assessment of QoL to address an important question for early-stage PC.

## CONCLUSIONS AND CLINICAL IMPLICATIONS

In conclusion, EORTC QLQ-C30 and QLQ-PAN26 are the most commonly used PROMs for assessing QoL in patients with early-stage PC who are undergoing surgery. The poor EORTC QLQ-C30 GHS/QoL scores in PC compared with scores in all cancers indicate a high unmet need in this patient population. Although the aforementioned limitations, especially survival bias, should be considered, QoL declined immediately after surgery, recovered in approximately 3 to 6 months, and remained generally stable for the rest of the follow-up period. The MID values for QLQ-C30 may help elucidate the clinically relevant impact on QoL of treating early-stage PC. Future research should establish the MID for EORTC QLQ-PAN26 in this patient population.

The results of this and other studies reveal QoL patterns in patients with early-stage PC who underwent surgical resection. With this knowledge, physicians might be able to identify points of intervention through several approaches: symptom(s) management, psychological and social support, neoadjuvant therapy, and adjuvant therapy initiation as early as possible depending on the individual patient situation and opinion of the treating physician. Collectively, a holistic approach to QoL management may help further refine the treatment guidelines in this patient population.

## Supplementary Material

SUPPLEMENTARY MATERIAL
