# Supplementary material for: Landscape of Health-Related Quality of Life in Patients With Early-Stage Pancreatic Cancer Receiving Adjuvant or Neoadjuvant Chemotherapy: A Systematic Literature Review
Source: Pancreas. 2019 Mar 10;49(3):393–407. doi: 10.1097/MPA.0000000000001507 (PMC7077976; doi:10.1097/MPA.0000000000001507)
Supplement: SUPPLEMENTARY MATERIAL [file mpa-49-393-s001.pdf]

Landscape of Health-Related Quality of Life in Patients With Early-Stage Pancreatic Cancer Receiving Adjuvant or Neoadjuvant Chemotherapy

A Systematic Literature Review

SUPPLEMENTAL DIGITAL CONTENT

SUPPLEMENTAL TABLE 1. Search Syntax

Item	Search Terms	Included in General Search	Included in Supplemental Search
**Population**			
Disease (pancreatic cancer mesh terms OR resection and pancreatic cancer key words)	(EMB.EXPLODE("pancreas cancer") OR MESH.EXPLODE("Pancreatic Neoplasms") OR EMB.EXPLODE("pancreas resection") OR "pancreas resection" OR (resect* AND ("pancreatic cancer" OR "pancreas cancer" OR "pancreas resection" OR "pancreas surgery")))	Y	Y
**Intervention**			
Adjuvant therapy	(MESH.EXACT("Chemotherapy, Adjuvant") OR EMB.EXACT.EXPLODE("cancer adjuvant therapy") OR EMB.EXACT.EXPLODE("adjuvant therapy") OR TI,AB(adjuvant OR "adjuvant therapy" OR "adjuvant cancer therapy" OR "adjuvant chemotherapy"))	Y	N
Neoadjuvant therapy	("Neo-adjuvant" OR "Neo adjuvant" OR "Neoadjuvant")	Y	N
**Comparison**			
NA		NA	NA
**Outcome**			
EORTC QLQ-PAN26	TI,AB("EORTC QLQ-PAN26" OR "EORTC QLQ PAN26" OR "QLQ-PAN26" OR "QLQ PAN26" OR "QLQPan26" OR "Pan 26" OR "Pan26")	N	Y
QoL	EMB.EXPLODE("patient-reported outcome") OR MESH.EXPLODE("Patient Reported Outcome Measures") OR MESH.EXPLODE("Quality of Life") OR EMB.EXPLODE("quality of life") OR EMB.EXPLODE("quality of life assessment") OR TI,AB("health related quality of life" OR "HRQOL" OR "HQL" OR "HQOL" OR "HR-QOL" OR "QOL" OR "quality of life" OR "patient reported outcomes" OR "patient reported outcome" OR "patient reported" OR "patient-reported" OR "patient satisfaction" OR "quality of well being" OR "quality of wellbeing" OR "quality of well-being" OR "QWB")	Y	N

(Continued on next page)

SUPPLEMENTAL TABLE 1. *(Continued)*

Item	Search Terms	Included in General Search	Included in Supplemental Search
MID	EMB.EXPLODE("minimal clinically important difference") OR MESH.EXPLODE("Minimal Clinically Important Difference") OR "minimally important difference" OR “minimal important difference” OR "minimal clinically important difference" OR "clinically meaningful change" OR “half standard deviation” OR “1/2 standard deviation” OR “standard error of measurement” OR “anchor-based method” OR “anchor based method” OR “responder” OR “responder analysis” OR “response analysis” OR “responder definition” OR “response definition” OR “clinical responder” OR “clinical response”	Y	N
**Setting**			
NA*		NA	NA

*Studies not limited by clinical trial to obtain comprehensive evidence.

EORTC indicates European Organisation for Research and Treatment; HRQOL, health-related quality of life; QLQ-PAN26, Quality of Life questionnaire-pancreatic cancer; QoL, quality of life; QWB, quality of well-being; MID, minimally important difference; NA, not applicable.
